# Outcome prediction in aneurysmal subarachnoid hemorrhage with world federation of neurological societies grade V (OPAS-V)

**DOI:** 10.1007/s00701-025-06611-7

**Published:** 2025-07-24

**Authors:** Shuhei Yamada, Takeo Nishida, Tomofumi Takenaka, Hiroki Yamazaki, Ryota Nakagawa, Masatoshi Takagaki, Yoshihiro Yano, Hajime Nakamura, Shingo Toyota, Toshiyuki Fujinaka, Takuyu Taki, Toshiaki Fujita, Haruhiko Kishima

**Affiliations:** 1 Department of Neurosurgery, the University of Osaka Graduate School of Medicine, 2-2 Yamadaoka, Suita, Osaka, 565-0871 Japan; 2 Department of Neurosurgery, Kansai Rosai Hospital, Amagasaki, Hyogo Japan; 3 Department of Neurosurgery, National Hospital Organization Osaka National Hospital, Osaka, Japan; 4 Department of Neurosurgery, Hanwa Memorial Hospital, Osaka, Japan

**Keywords:** Subarachnoid hemorrhage, WFNS grade V, Prognosis, Prediction, Multicenter study

## Abstract

**Background:**

Aneurysmal subarachnoid hemorrhage (aSAH) with World Federation of Neurological Societies (WFNS) grade V has a high mortality rate and poor prognosis. Some patients with WFNS grade V aSAH have had good outcomes after aggressive treatment; however, outcome predictions based on routine examinations and findings obtained at admission are yet to be reported. This study aimed to develop a decision tree model for predicting outcomes of patients with WFNS grade V aSAH to aid decision-making for treatment strategy.

**Method:**

A multicenter study with retrospective and prospective data collected from 201 (derivation cohort) and 26 (validation cohort) patients with WFNS grade V aSAH, respectively, was conducted. Clinical outcomes were divided into good (Modified Rankin Scale [mRS] score at the time of discharge: 0–2) and poor (mRS score: 3–6) outcomes. A decision tree model was developed for the derivation cohort using the classification and regression tree method with clinical data including laboratory findings; it was named OPAS-V (Outcome Prediction in Aneurysmal Subarachnoid hemorrhage with WFNS grade V). The performance of the model was evaluated by area under the curve (AUC) and overall accuracy in both cohorts.

**Results:**

OPAS-V comprised 3 metrics; the percentage of lymphocytes (< 49.9% or not), age (> 50 yrs or not), and glucose to potassium ratio (≥ 3.2 or not). The model achieved an AUC of 0.828 (95% confidence interval: 0.712–0.944) and overall accuracy of 0.930. Moreover, the model performed well in the validation cohort with an AUC of 0.727 (95% confidence interval: 0.441–1) and overall accuracy of 0.885.

**Conclusions:**

This study developed the first decision tree model for predicting outcomes of patients with WFNS grade V aSAH, based on simple findings obtained at admission. This may aid clinicians in determining treatment strategies for severe conditions such as WFNS grade V aSAH.

**Supplementary information:**

The online version contains supplementary material available at 10.1007/s00701-025-06611-7.

## Introduction

Aneurysmal subarachnoid hemorrhage (aSAH) causes severe neurological deficit and mortality, despite recent progress in treatment [25]. The World Federation of Neurological Societies (WFNS) Scale has been the most significant outcome predictor in several large-scale prognostic studies conducted on patients with aSAH [13, 30, 42]. Recent studies focusing on patients with poor-grade aSAH have also revealed a clear impact of WFNS grade V on their predicted outcomes [23, 33].

WFNS grade V aSAH accounts for 20–35% of all cases [28, 40, 41, 43], and has a high mortality rate of 48–63% during hospitalization [28, 45]. Conversely, several reports have revealed that 14–25% of aggressively treated patients with WFNS grade V aSAH had good outcomes [1, 17, 35, 41, 45]. The clinical severity of aSAH at admission has been reported to reflect the acute inflammatory response, including early brain injury (EBI) [11, 14, 29]. In patients with WFNS grade V, the impact of EBI on their outcomes may be heterogeneous[29, 37] and should be considered when developing outcome prediction models.

Laboratory findings are usually reviewed at admission for aSAH as well as physical and radiological examinations. Many studies have reported that specific blood test findings at admission correlate with EBI or outcomes after aSAH[3, 8, 10, 14, 18, 21, 22, 26, 27, 31, 37–39, 47, 49] However, no previous study has successfully predicted the outcome in patients with WFNS grade V aSAH, based on the routine examinations and findings obtained at admission, including these laboratory findings.

This multicenter study aimed to develop a decision tree model for predicting outcomes of patients with WFNS grade V aSAH using only routine assessment findings obtained at admission to aid decision-making for treatment strategy.

## Methods and materials

### Study design and patient selection

We conducted a multicenter study of patients with severe aSAH, defined as grade V, according to the WFNS scale. We collected retrospective data from patients who were admitted to 4 institutions (Osaka University Hospital, Osaka, Japan; Kansai Rosai Hospital, Hyogo, Japan; Hanwa Memorial Hospital, Osaka, Japan; and Osaka National Hospital, Osaka, Japan) between August 2012 and June 2021, for the derivation cohort. We defined the validation cohort as prospective data from patients who were admitted to the first three of these institutions between July 2021 and September 2023. We treated all patients with WFNS grade V aSAH aggressively, except those with unstable vital signs or those with increased intracranial pressure that prevented the contrast from entering the intracranial space. The inclusion criteria were as follows: (1) patients diagnosed with SAH using computed tomography (CT); (2) patients with saccular aneurysms confirmed via three-dimensional CT angiography (CTA) or digital subtraction angiography at admission; (3) patients with WFNS grade V; (4) patients who underwent surgical intervention (craniotomy and/or endovascular treatment) for aneurysmal occlusion; and (5) patients whose family members provided informed consent for surgical intervention. We excluded patients with a pre-stroke Modified Rankin Scale (mRS) score ≥ 3.

### Outcomes

We assessed clinical outcomes using mRS score at the time of discharge. We defined good and poor outcomes as mRS scores of 0–2 and 3–6, respectively [3, 35].

### Clinical data collection

We retrospectively collected patients’ characteristics from the medical records. The lead author (S.Y.) carefully determined radiological findings (for example, Fisher group[6] or modified Fisher grade[7]) via initial CT, which was independently confirmed by the senior author (T.N.). We collected the initial laboratory findings at admission or within the initial 24 h after admission, and calculated the neutrophil to lymphocyte ratio (NLR) [3, 10, 38], glucose to potassium ratio [8], and C-reactive protein (CRP) to albumin ratio [49]. We used SI units for the laboratory findings [48].

### Decision tree model development

We used the classification and regression tree (CART) method as the learning algorithm [2]. The CART method is nonparametric and does not depend on a specific type of data distribution,[9]; hence, we included all variables in the CART analysis. We substituted missing values with the most frequent value and median value for categorical and continuous variables, respectively. We evaluated the performance of a decision tree model by area under the curve (AUC) of receiver operating characteristic (ROC) analysis, overall accuracy, sensitivity, and specificity. Overall accuracy was the ratio of correctly predicted outcomes. Sensitivity and specificity were the ratios of the predicted good or poor outcomes, respectively, that had actually developed.

### Human ethics and consent to participate

The Osaka University Clinical Research Review Committee approved the study (approval number 19486) and waived the need for additional written informed consent. All procedures performed in this study including those involving human participants were in accordance with the ethical standards laid down in the 1964 Decleration of Helsinki and its later amendments. This study adhered to the STROBE (Strengthening the Reporting of Observational Studies in Epidemiology) reporting guideline [44].

### Data availability

The anonymized data that support the findings of this study are available from the corresponding author upon reasonable request.

### Statistical analysis

All statistical analyses were performed using R 4.1.1 for Windows (www.R-project.org; R Foundation for Statistical Computing, Vienna, Austria). Categorical variables were assessed using Fisher’s exact test and presented as frequency (percentages). Continuous variables were assessed using the Mann–Whitney U test and presented as median and interquartile range (IQR). Multivariate logistic regression analysis was performed with variables that were significant in the univariate analyses. Statistical significance was set at *p* < 0.05.

## Results

### Patient characteristics and outcomes

In the derivation cohort, 210 patients with WFNS grade V aSAH underwent surgical intervention for aneurysmal occlusion. Among them, 9 were excluded because of their poor pre-stroke mRS score ≥ 3. Thus, the data of 201 (67 male and 134 female) and 26 (6 male and 20 female) patients in the derivation and validation cohorts, respectively, were independently analyzed. Patient characteristics are presented in Table 1. The median age at aSAH onset was 70 (IQR: 58–79 years) and 62 (IQR: 54–75 years) years in the derivation and validation cohorts, respectively.

**Table 1:** Patient characteristics

Variable n (%)/median (IQR)	Derivation cohort *n* = 201	Validation cohort *n* = 26
Baseline Characteristics		
Sex (Female)	134 (67%)	20 (77%)
Age (years)	70 (58–79)	62 (54–75)
Aneurysm side		
Left	74 (37%)	10 (38%)
Midline	57 (28%)	9 (35%)
Right	70 (35%)	7 (27%)
Aneurysm location (anterior circulation)	183 (91%)	24 (92%)
Pupillary status ^a^		
Isocoria	131 (65%)	21 (81%)
Anisocoria	40 (20%)	1 (4%)
Fixed dilated	29 (14%)	3 (12%)
Pre-stroke mRS		
0	194 (97%)	23 (88%)
1	1 (0%)	2 (8%)
2	6 (3%)	1 (4%)
Hospital duration (days)	47 (28–71)	42 (23–61)
mRS at discharge		
0–2	18 (9%)	4 (15%)
0–4	57 (28%)	9 (35%)
Patient history		
Hypertension	78 (39%)	10 (38%)
Diabetes mellitus	17 (8%)	1 (4%)
Dyslipidemia	20 (10%)	3 (12%)
Past subarachnoid hemorrhage	7 (3%)	1 (4%)
Smoking	24 (12%)	1 (4%)
Family history		
Subarachnoid hemorrhage	7 (3%)	2 (8%)
Radiological findings		
Fisher group		
2	4 (2%)	0 (0%)
3	194 (97%)	26 (100%)
4	3 (1%)	0 (0%)
Modified Fisher grade		
1	2 (1%)	0 (0%)
2	5 (2%)	0 (0%)
3	53 (26%)	9 (35%)
4	141 (70%)	17 (65%)
Intraventricular hemorrhage	145 (72%)	17 (65%)
Intracerebral hemorrhage	85 (42%)	16 (62%)
Laboratory findings		
WBC (10^9^/L)	12.6 (9.4–16.2)	11.9 (8.8–17.1)
Neutrophil (%) ^b^	79.6 (55.7–88.2)	85.5 (68.7–91.3)
Lymphocyte (%) ^b^	14.0 (7.0–35.3)	9.5 (4.3–23.5)
Monocyte (%) ^b^	4.6 (3.4–6.3)	4.1 (3.6–4.7)
NLR ^b^	5.64 (1.59–12.71)	9.00 (2.94–21.63)
Hemoglobin (g/L)	130 (118–143)	126 (115–141)
Hematocrit (/L)	0.40 (0.36–0.43)	0.37 (0.34–0.42)
PT (%)	99 (90–105)	93 (87–100)
PT-INR	1.00 (0.94–1.05)	1.00 (0.96–1.06)
APTT (sec) ^c^	26.4 (25.0–29.0)	27.0 (24.2–28.8)
D-dimer (mg/L) ^d^	4.44 (1.87–8.80)	5.53 (2.24–8.81)
FDP (mg/L) ^e^	13.8 (6.2–38.6)	17.8 (5.8–22.0)
Fibrinogen (g/L) ^f^	3.22 (2.72–3.65)	3.34 (2.56–3.85)
Na (mmol/L)	140 (138–142)	141 (139–141)
K (mmol/L)	3.3 (3.0–3.7)	3.4 (3.2–3.7)
Cl (mmol/L)	103 (101–105)	104 (102–107)
Glucose (mmol/L) ^g^	10.5 (9.2–12.5)	10.2 (8.4–10.9)
Glucose/K	3.1 (2.7–3.9)	2.7 (2.5–3.3)
BUN (mmol/L)	5.6 (4.6–6.8)	6.8 (5.5–8.7)
Creatinine (μmol/L)	61 (49–75)	66 (52–91)
Total protein (g/L) ^h^	73 (68–77)	71 (68–77)
Albumin (g/L)	41 (38–44)	38 (35–42)
CRP (mg/L)	1.0 (1.0–3.0)	1.0 (0.4–3.5)
CRP/Albumin	0.026 (0.021–0.079)	0.025 (0.013–0.078)

*mRS*; Modified Rankin Scale, *WBC*; White blood cell, *NLR*; Neutrophil–Lymphocyte ratio, *PT*; Prothrombin time, *PT-INR*; Prothrombin time-international normalized ratio, *APTT*; Activated partial thromboplastin time, *FDP*; Fibrinogen degradation products, *BUN*; Blood urea nitrogen, *CRP*; C-reactive protein

^a^ Two missing values

^b^ Eighty-one missing values

^c^ Twenty-eight missing values

^d^ Forty-two missing values

^e^ One hundred twenty-eight missing values

^f^ Forty-nine missing values

^g^ Fifteen missing values

^h^ Four missing values

The median duration of hospital stay was 47 (IQR: 28–71 days) and 42 (IQR: 23–61 days) days in the derivation and validation cohorts, respectively. Eighteen (9%) and 4 (15%) patients in the derivation and validation cohorts, respectively, had good outcomes (mRS score 0–2).

### Decision tree model distinguishing between mRS Score 0–2 and 3–6

**Fig. 1:**
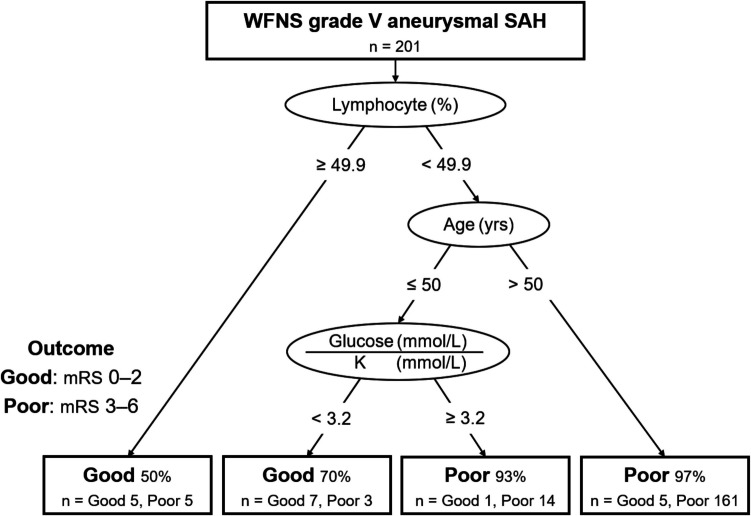
Outcome Prediction in Aneurysmal subarachnoid hemorrhage with World Federation of Neurological Societies grade V (OPAS-V). We have defined good and poor outcomes as modified Rankin Scale scores of 0–2 and 3–6, respectively, at the time of discharge. OPAS-V comprised 3 metrics; the percentage of lymphocytes, age, and glucose to potassium ratio.K: potassium, WFNS: World Federation of Neurological Societies, SAH: subarachnoid hemorrhage

We developed a decision tree model, termed OPAS-V (Outcome Prediction in Aneurysmal Subarachnoid hemorrhage with World Federation of Neurological Societies grade V), to distinguish between mRS score 0–2 (good outcome) and 3–6 (poor outcome) using the data of the derivation cohort (Fig. 1). OPAS-V consisted of 3 metrics; the percentage of lymphocytes, age, and glucose to potassium ratio. If the percentage of lymphocytes was > 49.9% at the first node, the patient had a good outcome (50%). If not, the next node was age; the patient had a poor outcome (97%), if > 50 years old. If not, the last node was the glucose to potassium ratio. If it was < 3.2, the patient had a good outcome (70%), and if not, a poor outcome (93%). OPAS-V achieved an AUC of 0.828 (95% confidence interval [CI]: 0.712–0.944), overall accuracy of 0.930, sensitivity of 0.667, and specificity of 0.956.

Furthermore, OPAS-V performed well in the validation cohort with an AUC of 0.727 (95% CI: 0.441–1), overall accuracy of 0.885, sensitivity of 0.500, and specificity of 0.955.

### Comparison of outcomes in the derivation cohort

Univariate analysis revealed significant differences between the outcomes (mRS score 0–2 and 3–6) in the derivation cohort in terms of age (*p* < 0.001), Fisher group (*p* = 0.009), modified Fisher grade (*p* = 0.018), intracerebral hemorrhage (ICH) (*p* = 0.005), percentage of neutrophils (*p* = 0.001), percentage of lymphocytes (*p* = 0.002), NLR (*p* = 0.002), and blood urea nitrogen (*p* = 0.042) (Supplementary Table 1). For multivariate analysis, Fisher group was excluded as it is similar to modified Fisher grade. Additionally, the percentage of neutrophils and NLR were excluded as they are related to the percentage of lymphocytes. Multivariate analysis for a poor outcome (mRS score 3–6) revealed significant differences in terms of age (*p* = 0.001, odds ratio [OR]: 1.08 [95% CI: 1.03–1.14]), ICH (*p* = 0.005, OR: 17.37 [95% CI: 2.39–126.34]), and the percentage of lymphocytes (*p* = 0.013, OR: 0.96 [95% CI: 0.92–0.99]) (Supplementary Table 2).

## Discussion

We have developed a decision tree model comprising simple findings obtained at admission, which accurately predict the outcome of patients with WFNS grade V aSAH and aid in clinical decision-making, including treatment strategies.

Maximal treatment for all patients with WFNS grade V aSAH is challenging and controversial due to high mortality rates and poor prognoses [17, 28]. Conversely, several reports have revealed that some of patients have good outcomes [1, 17, 35, 41, 45]. It is still unclear which factors develops this heterogeneity in their outcomes. However, our decision tree model, OPAS-V, based on the routine examinations including laboratory findings at admission may be useful in identifying patients who need aggressive treatment. This may be because the laboratory findings included in OPAS-V indicate a certain impact of EBI on the outcome of patients with aSAH. Further external validation is needed because of the small number of patients in the validation cohort.

OPAS-V comprised 3 metrics; the percentage of lymphocytes, age, and glucose to potassium ratio. The nodes derived by CART analysis are not necessarily known predictors. However, age was a correlated metric with a good outcome at the time of discharge [12], as confirmed in this study. The glucose to potassium ratio and NLR, which is related to the acute inflammatory response and percentage of lymphocytes, have also frequently been reported to be correlated with aSAH and other acute brain injury-related diseases, in recent years [3, 8, 10, 14, 20, 27, 32, 38, 46, 50]. Further studies should focus on laboratory findings, including these indices, in patients with WFNS grade V aSAH.

Few prognostic models have considered laboratory findings obtained at admission, in addition to physical and radiological findings [21]. Our model is applicable even to WFNS grade V because many clinicians routinely obtain the components of our model during patient assessment at admission. The Glasgow Coma Scale motor value on the second day of hospitalization or CTA source images at admission may impact the outcome of WFNS grade V aSAH [35, 41]. Furthermore, uncommon laboratory findings such as serum S100 and serum adipocyte fatty acid-binding proteins have been reported to correlate with the prognosis of aSAH [15, 24, 34]. However, these findings might be difficult for application in daily clinical practice.

The SAFIRE grading scale, a recent large-scale study including all WFNS grade patients, reported good discrimination with internal and external AUCs of 0.90 and 0.73, respectively [42]. However, WFNS grade V has > 50% risk of poor outcomes, and only insufficient subdivision has been achieved [42]. This is partly because the clinical course and functional prognosis are significantly correlated with the severity of aSAH [5, 12, 36]. Further studies focused on patients with WFNS grade V aSAH are required.

A recent report of poor-grade aSAH revealed an AUC of 0.844 and 0.831 in the derivation and validation cohorts, respectively [33]. However, delayed cerebral ischemia and shunt-dependent hydrocephalus, which are yet to be determined at admission, were included in the prognostic model [33]. Another study of poor-grade aSAH developed a decision tree model with internal and external AUCs of 0.88 and 0.94, respectively [23]. However, this decision tree was more complex than OPAS-V and included pupillary reactivity, which was not assessed using the standardized protocol, as mentioned in their limitation [23].

This study has some limitations. First, it was performed in Japan; hence the treatment strategy for WFNS grade V aSAH may differ from that in other countries [4, 5, 36]. Second, the outcome was set as mRS at the time of discharge. This may lead to overlooking patients who may have favorable outcomes a few years after discharge [1, 19]. Indeed, the percentage of patients with good outcomes in this study was lower than in other studies,[16, 19] which is a major challenge for the future. Third, patients who could not undergo surgical intervention for aneurysmal occlusion were omitted; however, they all died during hospitalization, as in a previous study [45]. It should be noted that OPAS-V may be helpful only in an aggressive treatment approach for patients with WFNS grade V aSAH. And last, it should also be noted that OPAS-V is the clinical prediction model and it is unclear whether improving the metrics that comprised it will lead to improved patients’ outcomes.

In conclusion, we have developed the first decision tree model, OPAS-V, to predict the outcome of patients with WFNS grade V aSAH based on the simple findings obtained at admission. Furthermore, OPAS-V comprised 3 metrics; the percentage of lymphocytes, age, and glucose to potassium ratio. This may help clinicians in determining treatment strategies for severe conditions such as WFNS grade V aSAH, at admission.

## Supplementary information

Below is the link to the electronic supplementary material.

ESM 1 (DOCX 51.9 KB)

## Data Availability

The anonymized data that support the findings of this study are available from the corresponding author upon reasonable request.
