# Supplementary material for: Outcome prediction in aneurysmal subarachnoid hemorrhage with world federation of neurological societies grade V (OPAS-V)
Source: Acta Neurochir (Wien). 2025 Jul 24;167(1):200. doi: 10.1007/s00701-025-06611-7 (PMC12287215; doi:10.1007/s00701-025-06611-7)
Supplement: Supplementary file 1 — (DOCX 51.9 KB) [file 701_2025_6611_MOESM1_ESM.docx]

**Supplementary Tables**

Table 1. Comparison of outcomes in the derivation cohort

| Variable  n (%) / median (IQR) | Good outcome (mRS score: 0–2)  n = 18 (9%) | Poor outcome (mRS score: 3–6)  n = 183 (91%) | *p*-value |
| --- | --- | --- | --- |
| **Baseline Characteristics** |  |  |  |
| Sex (Female) | 14 (78%) | 120 (66%) | 0.433 |
| Age (years) | 55 (46–64) | 71 (60–79) | < 0.001 |
| Aneurysm side |  |  | 0.526 |
| Left | 9 (50%) | 65 (36%) |  |
| Midline | 4 (22%) | 53 (29%) |  |
| Right | 5 (28%) | 65 (36%) |  |
| Aneurysm location (anterior circulation) | 16 (89%) | 167 (91%) | 0.667 |
| Pupillary status ^a^ |  |  | 0.346 |
| Isocoria | 15 (83%) | 116 (63%) |  |
| Anisocoria | 2 (11%) | 38 (21%) |  |
| Fixed dilated | 1 (6%) | 28 (15%) |  |
| Pre-stroke mRS |  |  | 1 |
| 0 | 18 (100%) | 176 (96%) |  |
| 1 | 0 (0%) | 1 (1%) |  |
| 2 | 0 (0%) | 6 (3%) |  |
| **Patient history** |  |  |  |
| Hypertension | 5 (28%) | 73 (40%) | 0.448 |
| Diabetes mellitus | 0 (0%) | 17 (9%) | 0.373 |
| Dyslipidemia | 3 (17%) | 17 (9%) | 0.398 |
| Past subarachnoid hemorrhage | 0 (0%) | 7 (4%) | 1 |
| Smoking | 2 (11%) | 22 (12%) | 1 |
| **Family history** |  |  |  |
| Subarachnoid hemorrhage | 0 (0%) | 7 (4%) | 1 |
| **Radiological findings** |  |  |  |
| Fisher group |  |  | 0.009 |
| 2 | 1 (6%) | 3 (2%) |  |
| 3 | 15 (83%) | 179 (98%) |  |
| 4 | 2 (11%) | 1 (1%) |  |
| Modified Fisher grade |  |  | 0.018 |
| 1 | 1 (6%) | 1 (1%) |  |
| 2 | 2 (11%) | 3 (2%) |  |
| 3 | 6 (33%) | 47 (26%) |  |
| 4 | 9 (50%) | 132 (72%) |  |
| Intraventricular hemorrhage | 10 (56%) | 135 (74%) | 0.107 |
| Intracerebral hemorrhage | 2 (11%) | 83 (45%) | 0.005 |
| **Laboratory findings** |  |  |  |
| WBC (10^9^/L) | 11.8 (9.4–12.9) | 13.0 (9.4–16.3) | 0.171 |
| Neutrophil (%) ^b^ | 44.9 (38.2–66.5) | 80.9 (61.0–88.3) | 0.001 |
| Lymphocyte (%) ^b^ | 48.2 (26.2–53.6) | 12.8 (6.6–32.7) | 0.002 |
| Monocyte (%) ^b^ | 6.2 (4.7–6.7) | 4.6 (3.3–6.1) | 0.124 |
| NLR ^b^ | 0.93 (0.71–2.78) | 6.46 (1.87–13.33) | 0.002 |
| Hemoglobin (g/L) | 130 (124–133) | 133 (118–143) | 0.537 |
| Hematocrit (/L) | 0.39 (0.38–0.40) | 0.40 (0.36–0.43) | 0.707 |
| PT (%) | 100 (89–106) | 99 (90–105) | 0.892 |
| PT-INR | 0.99 (0.94–1.10) | 1.00 (0.94–1.05) | 0.753 |
| APTT (sec) ^c^ | 26.3 (25.5–27.7) | 26.6 (25.0–29.0) | 0.967 |
| D-dimer (mg/L) ^d^ | 2.84 (1.67–6.59) | 4.45 (1.89–8.83) | 0.376 |
| FDP (mg/L) ^e^ | 7.5 (6.5–18.4) | 14.0 (6.3–39.2) | 0.502 |
| Fibrinogen (g/L) ^f^ | 3.06 (2.43–3.48) | 3.24 (2.75–3.64) | 0.463 |
| Na (mmol/L) | 140 (138–142) | 140 (138–142) | 0.917 |
| K (mmol/L) | 3.3 (2.9–3.4) | 3.4 (3–3.7) | 0.100 |
| Cl (mmol/L) | 104 (102–105) | 103 (101–105) | 0.533 |
| Glucose (mmol/L) ^g^ | 9.7 (8.0–11.7) | 10.5 (9.2–12.6) | 0.069 |
| Glucose / K | 3.1 (2.6–3.6) | 3.1 (2.7–4.0) | 0.487 |
| BUN (mmol/L) | 4.8 (4.3–5.7) | 5.7 (4.6–6.8) | 0.042 |
| Creatinine (μmol/L) | 57 (45–65) | 61 (49–77) | 0.218 |
| Total protein (g/L) ^h^ | 72 (69–75) | 73 (68–77) | 0.811 |
| Albumin (g/L) | 41 (39–43) | 41 (38–44) | 0.900 |
| CRP (mg/L) | 1.0 (1.0–3.0) | 1.0 (0.9–3.0) | 0.740 |
| CRP / Albumin | 0.028 (0.024–0.066) | 0.026 (0.021–0.080) | 0.742 |

mRS: Modified Rankin Scale

WBC: White blood cell

NLR: Neutrophil-Lymphocyte ratio

PT: Prothrombin time

PT-INR: Prothrombin time-international normalized ratio

APTT: Activated partial thromboplastin time

FDP: Fibrinogen degradation products

BUN: Blood urea nitrogen

CRP: C-reactive protein

^a^ One missing value

^b^ Seventy missing values

^c^ Twenty-eight missing values

^d^ Forty-one missing values

^e^ One hundred thirteen missing values

^f^ Forty-five missing values

^g^ Fifteen missing values

^h^ Four missing values

Table 2. Results of multiple logistic regression analysis for a poor outcome (mRS score: 3–6)

| Variable | *p*-value | Odds ratio (95% CI) |
| --- | --- | --- |
| Age | 0.001 | 1.08 (1.03–1.14) |
| Modified Fisher grade |  |  |
| 1 | - | 1.0 (reference) |
| 2 | 0.414 | 0.19 (0.004–9.96) |
| 3 | 0.326 | 5.22 (0.19–141.50) |
| 4 | 0.198 | 8.51 (0.33–221.45) |
| Intracerebral hemorrhage |  |  |
| No | - | 1.0 (reference) |
| Yes | 0.005 | 17.37 (2.39–126.34) |
| Lymphocyte (%) | 0.013 | 0.96 (0.92–0.99) |
| BUN | 0.237 | 1.10 (0.94–1.30) |

mRS: Modified Rankin Scale

CI: Confidence Interval

BUN: Blood urea nitrogen
